# A knowledge-driven unified framework for plant disease classification and severity grading via domain adaptation

**DOI:** 10.1016/j.plaphe.2026.100233

**Published:** 2026-06-06

**Authors:** Zhihao Yuan, Xingcai Wu, Yang Liu, Xiaomin Shuai, Xulong Huang, R.D.S.M. Gunarathna, Peijia Yu, Yuanyuan Xiao, Qi Wang

**Affiliations:** a Guizhou Provincial Laboratory of Big Data, College of Computer Science and Technology, Guizhou University, Guiyang, 550025, China; b School of Pharmacy, Chengdu University of Traditional Chinese Medicine, Chengdu, 611137, China; c Postgraduate Institute of Agriculture, University of Peradeniya, Peradeniya, 20400, Sri Lanka

**Keywords:** Plant disease diagnosis, Knowledge-driven learning, Domain adaptation

## Abstract

Plant leaf disease classification and severity grading are essential for precision agriculture, enabling timely intervention and optimized management. Existing models often fail to recognize previously unseen disease categories due to rigid label spaces and limited representation of plant phenotypes. To address these challenges, we propose a knowledge-driven unified framework for plant disease classification and severity grading. A Common Knowledge Learner consolidates fundamental features of plant species, disease categories, and severity levels from labeled data, forming a transferable representation space. It employs a multi-level contrastive learning strategy to capture both global semantic representations and fine-grained lesion patterns. Building on these representations, a Cross-Domain Adaptation module leverages a teacher–student framework with Low-Rank Adaptation (LoRA) bridges in-domain and out-of-domain feature spaces using large-scale unlabeled data. Meanwhile, a contrastive feature library enables similarity-based reasoning and supports flexible label space expansion during inference without retraining. We evaluate our approach on Leaf-CG, a large-scale dataset comprising 441,448 images from 59 plant species, 373 disease categories, and four severity levels. Experiments demonstrate that our framework outperforms existing baselines, achieving 94.9% disease classification accuracy and 90.6% severity grading accuracy in-domain. Under out-of-domain conditions, the method achieves 82.1% true positive rate (TPR) in open-set settings, highlighting its strong generalization ability and potential for practical plant disease management. Code and dataset are available at https://www.uniplantcg.samlab.cn.

## 1 Introduction

Plant diseases seriously threaten plant productivity, which is fundamental to global food security and agricultural sustainability. Previous studies report that plant diseases cause more than 30% of annual global crop yield losses and result in economic damages amounting to hundreds of billions of dollars worldwide [1,2]. To reduce these losses, effective plant disease identification plays a crucial role in supporting disease prevention and control. With the rapid development of deep learning techniques, automated plant disease recognition achieves substantial improvements in diagnostic accuracy and efficiency [[3], [4], [5]]. However, accurate disease category recognition alone can no longer fully satisfy the requirements of modern scientific plant protection, as it provides limited information for assessing disease progression and determining suitable control intensity. Therefore, there is a growing need for integrated approaches that jointly consider disease classification and severity grading to enable comprehensive plant disease management.

In the field of plant leaf disease classification and severity grading, numerous data-driven approaches demonstrate high levels of automation and diagnostic accuracy under controlled experimental conditions [[6], [7], [8]]. For example, Kundu et al. [6] develop a deep convolutional framework for plant disease recognition. Liu et al. [7] introduce a learning-based grading model that achieved competitive performance on benchmark datasets. Despite these advances, model performance is often adversely affected by variations in illumination, background complexity, and imaging devices when applied to real production scenarios [[9], [10], [11]]. In addition, practical applications often involve newly emerging crop varieties and disease types that are absent from existing training datasets. This further challenges the robustness and real-world applicability of current methods. Under such circumstances, phenotypic variability and previously unseen disease manifestations can lead to inaccurate identification, thereby limiting the effectiveness of automated approaches for plant disease management [[12], [13], [14]].

**Fig. 1:**
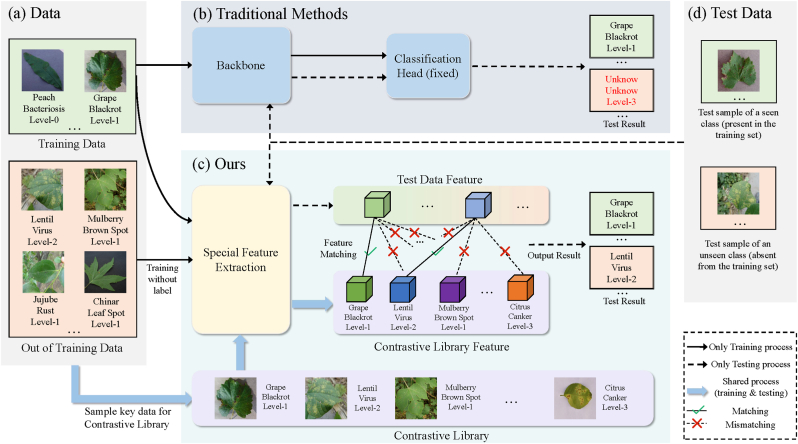
Overview of the proposed knowledge-guided plant disease classification and severity grading framework. (a) Training data with labels and out-of-training samples without labels are used for feature alignment. (b) Traditional methods extract features with a backbone and predict fixed categories using a classification head. (c) Our approach leverages special feature extraction and a contrastive knowledge library to perform knowledge-guided matching for both seen and unseen disease categories. (d) Test data include samples from seen and unseen classes for evaluation.

To mitigate these issues, subsequent studies explore domain adaptation [15] and domain generalization [16] techniques to enhance robustness under varying image acquisition conditions. For example, Giuffrida et al. [17] investigate domain adaptation strategies to reduce performance discrepancies between laboratory and field images. Batchuluun et al. [18] propose a segmentation-based approach to improve cross-domain generalization. Zhang et al. [19] propose EasyDAM_V3 for cross-species fruit detection and automatic labeling, where a multidimensional phenotypic feature space based on shape, color, and texture is constructed to select an optimal source domain, followed by target-domain image generation and synthetic dataset construction for downstream detector training. These studies mainly enhance cross-domain robustness through feature adaptation, segmentation-guided generalization, or synthetic target-domain data construction, but they generally assume predefined category spaces and do not address domain adaptation for previously unseen categories. Open-set recognition methods [[20], [21], [22]] enable the detection of samples from previously unseen categories during inference, as illustrated in Fig. 1b. However, existing open-set recognition methods typically assign all novel samples to a generic unknown class and fail to offer explicit disease or severity information. This limitation underscores the need for methods capable of accurately recognizing previously unseen plant diseases.

Phenotypic variability and the presence of previously unseen disease patterns pose persistent challenges to traditional plant disease classification and grading. Recent studies [23,24] indicate that Large Language Models (LLMs) [25] using Retrieval-Augmented Generation (RAG) [26] can effectively handle cases not encountered during training. This observation prompts us to reconsider conventional data-driven supervised training strategies. It also motivates the exploration of how richer structured information can be integrated into plant disease classification and grading to go beyond fixed label spaces and improve practical robustness. At a high level, this motivation is related to prior knowledge-guided transfer methods such as EasyDAM_V3 [19]. However, in our framework, knowledge supports recognition, while cross-domain adaptation is handled separately in Cross-Domain Adapter. As illustrated in Fig. 1c, incorporating a contrastive knowledge repository provides additional structured information for disease understanding. Through feature-level comparisons between query samples and reference representations, models are able to generalize to diverse and previously unseen phenotypes. As a result, recognition of varied disease manifestations and novel categories is enhanced, supporting comprehensive plant disease classification and severity grading. Unlike conventional data-driven classifiers that rely solely on supervised parameter learning, our framework explicitly organizes knowledge in an external contrastive repository, enabling flexible recognition and straightforward extension to new categories without retraining the backbone network.

In this paper, we propose a unified framework for plant leaf disease classification and severity grading, emphasizing robust generalization to real-world conditions. The framework builds upon a Contrastive Library that models plant species, disease categories, and severity levels in a flexible and decoupled manner. It incorporates a Common Knowledge Learning module trained on labeled data to acquire fundamental representations of plant species, diseases, and severity levels. A Cross-Domain Adaptation mechanism further exploits unlabeled samples beyond the training distribution, enabling effective generalization to unseen data without additional annotations. For comprehensive evaluation, we construct a large-scale, carefully annotated dataset containing 441,488 images from 59 plant species and 373 disease types, each annotated across four severity levels. Compared with existing plant disease datasets, this dataset offers substantially greater scale and diversity, providing a strong foundation for future research. Our main contributions are summarized as follows:

• We propose a unified domain generalization framework that assigns labels to unseen plant species and diseases using a knowledge-enhanced approach for robust performance.
• We develop a two-stage adaptation-and-inference scheme, where Cross Domain Adapter (CDA) enhances robustness under out-of-domain conditions, and the contrastive library enables novel-category recognition through similarity-based inference.
• We construct a large-scale, annotated dataset for plant leaf disease classification and severity grading, establishing a new benchmark and knowledge base.
• We conduct extensive experiments on both in-domain and out-of-domain datasets, demonstrating the framework's generalization ability and knowledge utilization.

## 2 Materials and methods

### 2.1 Datasets

Accurate and reliable assessment of plant leaf disease severity requires large-scale datasets covering multiple plant species and disease types, which are critical for modeling real-world complexity and diversity. However, most existing datasets remain limited to classification without severity annotations. For instance, mid-sized datasets such as AI Challenger 2018 [27] and PlantVillage [28], contain 50,000 and 54,309 images, covering 10 and 38 plant species as well as 27 and 152 disease types. Larger datasets further expand coverage. PDDD [29] includes 400,000 images across 120 disease categories from 40 plant species, and IP102 [30] provides 75,000 images covering 102 pest and disease categories from 8 plant species. In contrast, PlantDoc [31] focuses on real-world field conditions, comprising 2598 images of 17 diseases affecting 13 crops. Although these datasets provide valuable resources for plant disease classification research, they lack severity annotations and are therefore not suitable for disease grading tasks. To address this limitation, we construct a new plant leaf disease severity dataset, namely the Leaf-CG Dataset, which serves as the foundation for our study. Building on this dataset, we also establish a Contrastive Library to enable feature-level comparisons and facilitate robust classification and grading.

#### 2.1.1 Leaf-CG dataset

**Fig. 2:**
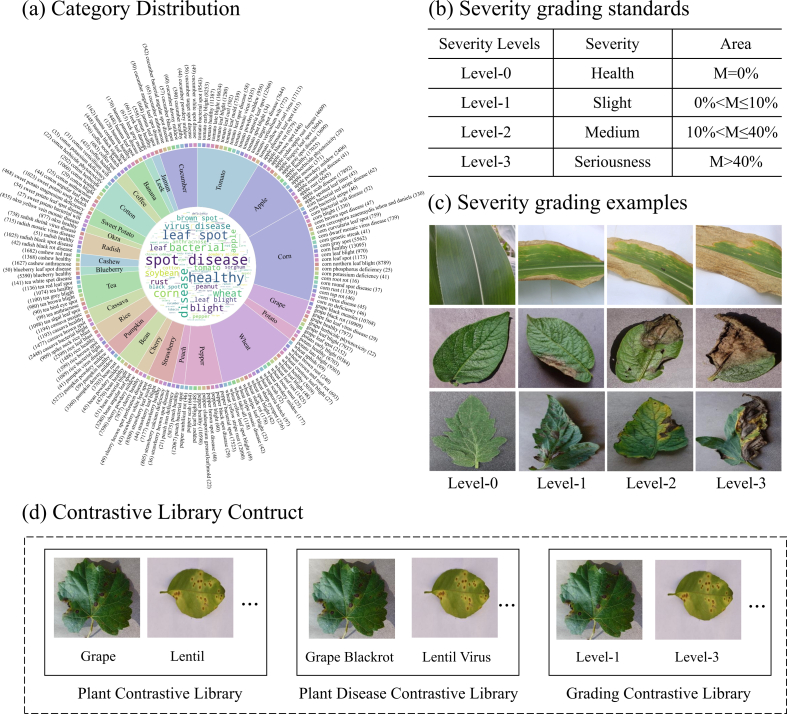
Ilustration of the proposed disease severity grading dataset. (a) Categories included in the dataset; (b) severity grading criteria; (c) examples of images in the constructed graded dataset; (d) construction of the contrastive feature libraries, where representative images are extracted and shared across three libraries with consistent images but different labels to support multiple tasks.

Compared with existing plant leaf disease datasets, Leaf-CG stands out by simultaneously supporting disease classification and severity grading. It contains 441,488 images spanning 59 plant species and 373 disease categories, each annotated with a severity grade (Fig. 2a). With its combination of scale, diversity, and multi-task annotations, Leaf-CG makes a valuable contribution toward developing and evaluating robust models for plant disease research.

To establish a consistent and practical severity reference, we consider that previous studies and agricultural guidelines adopt various disease severity grading systems [[32], [33], [34]], ranging from coarse to fine-grained scales. Motivated by these observations, we establish a four-level disease severity grading standard (levels 0–3) in collaboration with agricultural experts and following authoritative agricultural guidelines [35]. The four grades correspond approximately to 0%, ≤ 10%, 10–40%, and >40% of infected leaf area. This scheme balances scientific rigor and operational simplicity, providing enough granularity to capture disease progression while keeping each level actionable for management decisions. For annotation quality control, at least three agricultural experts independently review/each image, and a combination of random inspection and cross-validation ensures annotation consistency and reliability(Fig. 2c).

#### 2.1.2 Contrastive library

Aiming to provide the model with additional structured knowledge and enable knowledge-driven feature comparisons, we construct the contrastive library by sampling representative images from the Leaf-CG dataset, as illustrated in Fig. 2d. Specifically, for each plant–disease–severity category, approximately 5% of the training images are randomly sampled in a severity-balanced manner to ensure proportional representation across different severity levels. These images are fixed and do not participate in model parameter optimization, serving only as reference instances for contrastive comparison during training and inference. Based on the same set of images, three contrastive libraries are established with different label schemas, namely a plant contrastive library, a disease contrastive library, and a severity grading contrastive library. Although the image content is identical across the three libraries, each library is associated with task-specific labels, enabling flexible adaptation to plant classification, disease recognition, and severity grading without modifying the model architecture.

### 2.2 Methods

#### 2.2.1 Overview

**Fig. 3:**
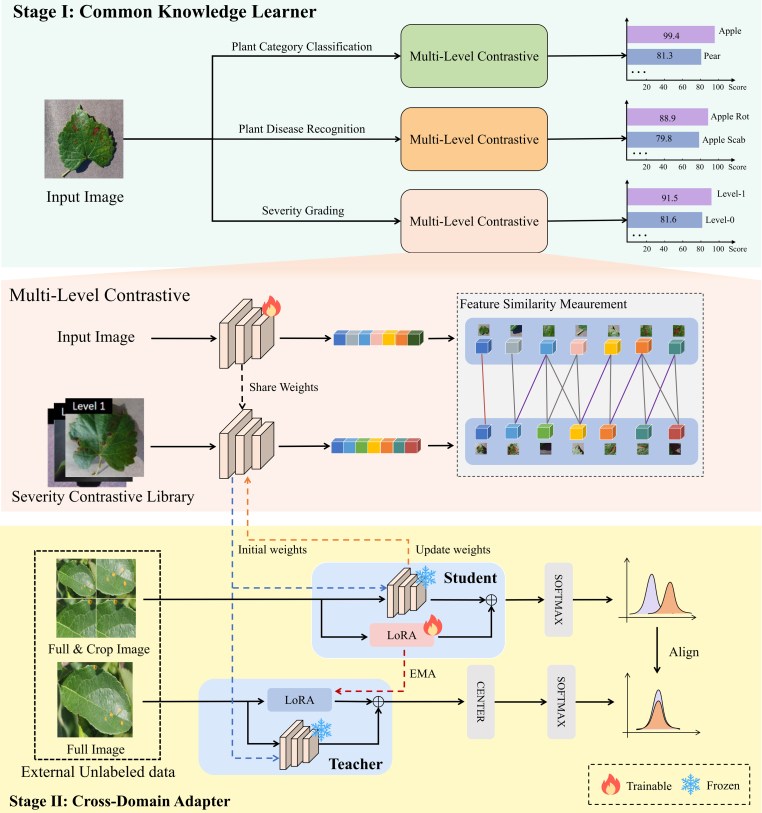
Overview of the proposed two-stage framework. Stage I: Common Knowledge Learner (CKL) consists of three parallel Multi-Level Contrastive (MLC) branches for plant classification, disease recognition, and severity grading, each matching image features against a task-specific labeled contrastive library. Stage II: Cross-Domain Adapter(CDA) improves generalization to unseen domains using external unlabeled data via a teacher–student framework, where the teacher is updated by Exponential Moving Average (EMA) and the student is adapted with Low-Rank Adaptation (LoRA) modules.

We propose a unified contrastive-based framework for plant leaf disease recognition and severity grading, as illustrated in Fig. 3. The framework comprises three key components: a Common Knowledge Learner(CKL), a Cross-Domain Adapter(CDA), and a contrastive feature library. Through multi-level contrastive learning on labeled data, the CKL captures fundamental representations of plant species, disease categories, and severity grading. To accommodate distribution shifts, the CDA exploits large-scale unlabeled out-of-domain data to adapt these representations to novel feature distributions. Built from labeled samples, the contrastive library serves as a reference for similarity-based reasoning, enabling flexible expansion of the label space during inference without retraining. Accordingly, cross-domain adaptation is performed in CDA, while the contrastive library mainly supports similarity-based recognition and scalable label-space extension during inference.

#### 2.2.2 Common Knowledge Learner

To equip the model with the basic ability to recognize plants, plant disease categories, and severity grading, we introduce the CKL as the main training stage of our plant leaf disease classification and severity grading framework (Fig. 3, Stage I). CKL processes individual diseased leaf images through task-specific feature encoders (plant classification, disease classification, and severity grading) combined with a contrastive learning strategy, and then infers task-specific attributes. Here, contrastive learning mainly serves as the representation-learning mechanism for organizing the embedding space in Stage I. Different from traditional classification methods based on fully connected layers with softmax outputs, CKL employs a contrastive matching mechanism built upon a contrastive feature library. This library contains diverse representative embeddings covering plant species, plant diseases, and severity levels, supporting robust alignment between input image features and library features. In this way, CKL improves generalization to novel categories and establishes a solid semantic foundation for subsequent CDA. This design reformulates recognition as similarity learning in an embedding space rather than direct decision-boundary optimization, which encourages the encoder to organize features according to semantic proximity.

At the core of CKL is the Multi-Level Contrast (MLC) module (Fig. 3). Unlike single-scale contrastive methods, MLC performs hierarchical contrastive matching between input image features and representative embeddings that reside in a contrastive feature library. By jointly constraining global semantic similarity and weighted local spatial similarity, MLC captures coarse disease patterns and fine-grained severity cues, thereby structuring the embedding space to maintain inter-class separation at the category level while preserving intra-class discrimination across severity variations. Specifically, given an input image **x**, a shared encoder *f*_*θ*_(⋅) extracts a set of embeddings

(1) $$Z=f_{\theta }\left(x\right)=\left\{z_{\mathrm{CLS}},z_{1},\ldots ,z_{N}\right\},$$

where **z**_*CLS*_ denotes the global semantic representation (CLS token), and **z**_*i*_ are local patch embeddings encoding fine-grained spatial information. To emphasize informative local regions, the MLC module assigns an adaptive importance weight to each local embedding:

(2) $$w_{i}=\frac{\exp\left(\phi \left(z_{i}\right)\right)}{\overset{N}{\underset{k=1}{\sum }}\exp\left(\phi \left(z_{k}\right)\right)},$$

where *ϕ*(⋅) is a learnable weighting function.

Algorithm 1 Multi-Level Contrastive(MLC)

**Table:** 

**Require:** Input image **x**; contrastive feature library C; balancing coefficient *α*
**Ensure:** Predicted label $\hat{y}$
1: Extract embeddings **Z** = {**z**_*CLS*_, **z**_1_, …, **z**_*N*_} from **x**
2: **for all**c∈C**do**
3: Contrastive Embeddings $Z^{c}=\left\{z_{\mathrm{CLS}}^{c},z_{1}^{c},\ldots ,z_{M}^{c}\right\}$
4: Compute global similarity:
5: $s_{\text{global}}\leftarrow \frac{z_{\mathrm{CLS}}\cdot z_{\mathrm{CLS}}^{c}}{\left\|z_{\mathrm{CLS}}\|\|z_{\mathrm{CLS}}^{c}\right\|}$
6: Compute local weights:
7: **for***i* = 1 to *N***do**
8: *e*_*i*_ ← *ϕ*(**z**_*i*_)
9: **end for**
10: *w*_*i*_ ←softmax(*e*_*i*_)
11: Compute local similarity:
12: *s*_local_ ← 0
13: **for***i* = 1 to *N***do**
14: **for***j* = 1 to *M***do**
15: $s_{\text{local}}\leftarrow s_{\text{local}}+w_{i}\cdot \frac{z_{i}\cdot z_{j}^{c}}{\left\|z_{i}\|\|z_{j}^{c}\right\|}$
16: **end for**
17: **end for**
18: Fuse similarities:
19: *s*_*c*_ ← *α* ⋅ *s*_global_ + (1 − *α*) ⋅ *s*_local_
20: **end for**
21: $\hat{y}\leftarrow \arg\max_{c\in C}s_{c}$

For each contrastive sample in the feature library, global semantic similarity and weighted local spatial similarity are jointly computed and fused into a unified similarity score. These similarity scores serve as implicit logits in the embedding space, enabling recognition to be performed through similarity-based probabilistic inference rather than fixed classifier outputs. During inference, the embedding set of the input image is exhaustively compared with those of all contrastive samples in the library. Algorithm 1 summarizes the complete multi-level similarity computation and matching procedure, where selecting the contrastive sample with the highest similarity determines the predicted label. The training objective combines a standard cross-entropy loss with a triplet-based contrastive loss:

(3) $$L_{\text{stage}1}=L_{\text{cls}}+\lambda \cdot L_{\text{triplet}},$$

where $L_{\text{cls}}$ supervises categorical prediction by applying softmax normalization over similarity scores, and $L_{\text{triplet}}$ enforces intra-class compactness and inter-class separability in the embedding space by imposing a margin constraint between anchor–positive and anchor–negative pairs, and *λ* is a balancing coefficient.

#### 2.2.3 Cross-Domain Adapter

To enhance the cross-domain generalization ability of the model, we propose a CDA based on a student–teacher framework, which leverages unlabeled out-of-domain data. The student–teacher paradigm performs self-distillation in the embedding space, encouraging the learning of domain-invariant representations without requiring target labels, while preserving knowledge acquired from in-domain categories. Specifically, we fine-tune the student network with Low-Rank Adaptation (LoRA), align its features with the teacher network using multi-scale local-to-global crops, and finally fuse the learned adaptations back into the backbone for inference.

In the first step, we denote the student and teacher networks $f_{\phi_{s}}$ and $f_{\phi_{t}}$ respectively, both initialized from the encoders trained in the first stage. To allow for effective domain adaptation while retaining prior knowledge, we introduce a LoRA layer in the student model. By restricting updates to low-rank adaptation matrices, the model adapts to distribution shifts in a parameter-efficient manner, reducing the risk of catastrophic forgetting of in-domain representations. The backbone weights *ϕ*_*s*_ and *ϕ*_*t*_ are frozen, while only the LoRA parameters Δ*ϕ*_*s*_ of the student are updated via gradient descent. The teacher's LoRA parameters Δ*ϕ*_*t*_ are updated using the Exponential Moving Average (EMA) of the student's LoRA parameters as:

(4) $$\Delta \phi_{t}\leftarrow \mu \;\Delta \phi_{t}+\left(1-\mu \right)\;\Delta \phi_{s},$$

where $\mu \in \left(\left[0,1\right)\right)$ is a momentum coefficient, which we set to 0.996 during training.

Subsequently, given an input image *u*, we generate a set of local crops $U=\left\{u_{1}^{s},\ldots ,u_{N}^{s}\right\}$ and two global crops $u_{1}^{t},u_{2}^{t}$. The student model processes all local crops, while the teacher only receives the global crops, forming a cross-view consistency learning scheme that aligns local and global representations across domains. Denoting the extracted features as:

(5) $$h_{i}^{s}=f_{\phi_{s}+\Delta \phi_{s}}\left(u_{i}^{s}\right),\;h_{j}^{t}=f_{\phi_{t}+\Delta \phi_{t}}\left(u_{j}^{t}\right),$$

we obtain probability distributions through temperature-scaled softmax:

(6) $$Q_{s}\left(u_{i}^{s}\right)\left(k\right)=\frac{\exp\left(h_{i}^{s}\left(k\right)/\tau_{s}\right)}{\sum_{k^{\prime }=1}^{K}\exp\left(h_{i}^{s}\left(k^{\prime }\right)/\tau_{s}\right)},$$

(7) $$Q_{t}\left(u_{j}^{t}\right)\left(k\right)=\frac{\exp\left(\left(h_{j}^{t}\left(k\right)+b\left(k\right)\right)/\tau_{t}\right)}{\sum_{k^{\prime }=1}^{K}\exp\left(\left(h_{j}^{t}\left(k^{\prime }\right)+b\left(k^{\prime }\right)\right)/\tau_{t}\right)},$$

where *τ*_*s*_, *τ*_*t*_ are temperature parameters for student and teacher, and $b\in R^{K}$ is a centering bias that regularizes the teacher's distribution.

To prevent feature collapse, the teacher's output distribution is regularized by maintaining a running mean of its logits:

(8) $$b\leftarrow \rho \cdot b+\left(1-\rho \right)\cdot \frac{1}{M}\overset{M}{\underset{i=1}{\sum }}f_{\phi_{t}+\Delta \phi_{t}}\left(u_{i}\right),$$

where *ρ* is the momentum coefficient and *M* is the batch size. This centering balances feature sharpness and uniformity, stabilizing the alignment process and implicitly constraining the entropy of the teacher distribution to avoid trivial solutions.

Finally, we fuse the teacher-side LoRA parameters into the backbone weights:

(9) $$\phi_{t}\leftarrow \phi_{t}+\Delta \phi_{t},$$

and load them back into the first-stage encoder. This ensures that the final model used during inference benefits from both the common knowledge and the domain-adaptive representations.

For each task branch, a KL-style alignment objective is introduced to minimize the discrepancy between cross-domain feature distributions, formulated as:

(10) $$L_{\text{align}}=-P_{t}\log P_{s},$$

where *P*_*t*_ and *P*_*s*_ denote the probability distributions or feature similarities produced by the teacher and student encoders, respectively.

#### 2.2.4 Training and inference strategy

The proposed model follows a two-stage training and inference strategy. The training process integrates CKL and CDA.The Inference process is evaluated on both in-domain categories (seen during training) and out-of-domain categories (unseen during training).

**Fig. 4:**
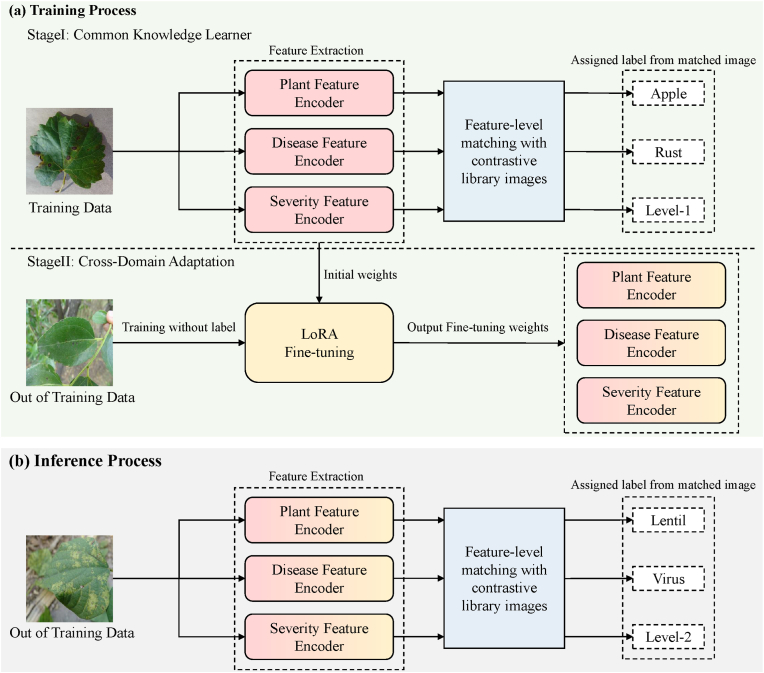
Overview of the training and inference pipeline. (a) The training process includes two stages: Common Knowledge Learner with labeled data and Cross-Domain Adaptationwith unlabeled data fine-tuning using LoRA. (b) During inference, features from out-of-domain images are matched against the contrastive library to assign predictions.

The CKL(see Fig. 4a) aims to establish robust and discriminative feature representations from well-annotated common categories, serving as a foundational model for subsequent cross-domain adaptation across plant species, disease types, and severity levels. All task branches (plant classification, disease recognition, and severity grading) share the same network architecture. The parameters of each branch are learned using supervised training, where each task has its own semantic labels. Cross-entropy loss updates the encoder's parameters, allowing the model to learn discriminative features for each task. This stage prepares the model for CDA, where the learned features are further adapted to unseen feature distributions.

The CDA(see Fig. 4a) aims to adapt the representations learned in CKL to broader and previously unseen feature distributions by leveraging large-scale unlabeled data, thereby enhancing the model's generalization ability to novel plant–disease categories. The encoder trained in CKL is fine-tuned on the unlabeled out-of-domain data. Using a student-teacher framework, the model adapts to the new feature distributions, improving its ability to align representations for novel classes in subsequent tasks. Since the data in CDA does not require labels, a wide range of data sources, including internet data, proprietary data, and public datasets, can be used for this stage, allowing the model to generalize to new domains effectively.

During inference (see Fig. 4b), the model is evaluated on both in-domain samples and out-of-domain samples consisting of entirely unseen plant–disease combinations. For each input image, features are extracted using the final encoder and matched against all features in the contrastive library processed by the same encoder. The label of the most similar contrastive image is assigned as the prediction. By progressively enriching the contrastive library with representative images, the output label space can be naturally expanded without retraining.

#### 2.2.5 Evaluation methods

To comprehensively evaluate the performance of the proposed method, we adopt Accuracy (ACC) and True Positive Rate (TPR) as the primary evaluation metrics. These metrics are selected to ensure a fair and consistent assessment under different learning settings.

ACC reflects the overall proportion of correctly classified samples among all test samples and is mainly used to evaluate supervised models under closed-set conditions. It is defined as:

(11) $$ACC=\frac{TP+TN}{TP+TN+FP+FN},$$

where *TP*, *TN*, *FP*, and *FN* denote the numbers of true positives, true negatives, false positives, and false negatives, respectively.

**Fig. 5:**
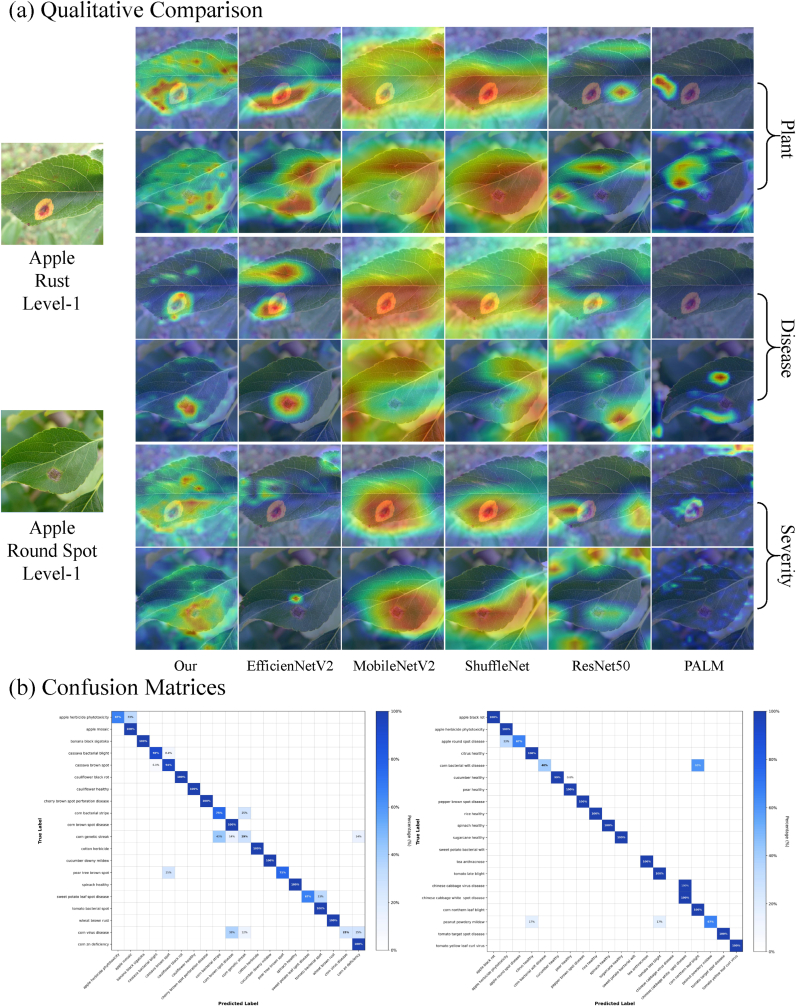
Visualization results of the proposed method. (a) Qualitative comparison with representative baseline models (EfficientNetV2, MobileNetV2, ShuffleNet, ResNet50 and PALM) on in-domain categories across three tasks: Plant (plant classification), Disease (disease recognition), and Severity (severity grading). (b) Confusion matrices of two randomly selected groups of 20 disease categories, where rows represent ground-truth labels and columns denote predicted labels. Diagonal elements indicate correctly classified samples.

In the multi-class classification setting, TP, TN, FP, and FN correspond to different regions of the confusion matrix shown in Fig. 5b, where rows represent the ground-truth labels and columns represent the predicted labels. For a given disease category, the diagonal element corresponds to correctly classified samples and therefore represents the True Positive (TP) cases for that category. For example, in Fig. 5b, the diagonal entries (dark blue cells) indicate that the majority of samples are correctly predicted as their true categories. Off-diagonal elements within the same row correspond to False Negatives (FN), where the true label belongs to a certain category but the model predicts another disease class. Conversely, off-diagonal elements within the same column correspond to False Positives (FP), where the model predicts a specific category while the true label belongs to a different class. Finally, True Negatives (TN) correspond to all samples that neither belong to the target category nor are predicted as that category, which are represented by all remaining cells outside the row and column associated with the target class. This interpretation ensures that the confusion matrix directly reflects the TP, TN, FP, and FN relationships used in the computation of ACC and TPR.

For traditional open-set models, overall accuracy may not adequately reflect the model's ability to correctly recognize known categories. Therefore, we additionally employ the TPR to evaluate performance in identifying known-class samples. TPR is defined as:

(12) $$TPR=\frac{TP}{TP+FN}.$$

Accordingly, ACC is reported for supervised models, while TPR is used for open-set models, ensuring appropriate and consistent evaluation across different model paradigms.

## 3 Experiments

### 3.1 Experimental setting and baselines

All experiments are implemented in Python 3.9.13 with PyTorch 2.3.1 and TorchVision 0.9.0. Training is conducted on a Linux 7.9 system with an NVIDIA A100 GPU (40 GB VRAM), an Intel Xeon Silver 4316 CPU, and 128 GB RAM. For training, all input images are resized to 256 × 256. The patch embedding layer adopts a 16 × 16 kernel size with stride 11 to generate overlapping patch tokens. The backbone network uses the GELU activation function. The model is optimized using SGD with weight decay. The initial learning rate is set to 0.0004, with a cosine annealing schedule applied over 150 training epochs. Additional hyperparameters, EMA momentum *μ* , temperature coefficients *τ*_*s*_ and *τ*_*t*_, are summarized in Table 1c.

**Table 1:** Overall performance evaluation of the proposed framework. (a) Comparison with supervised and open-set baselines across plant classification, disease recognition, and severity grading tasks under in-domain and out-of-domain settings. (b) Statistical validation results showing the mean ± standard deviation over three runs and paired *t*-test significance against the strongest baselines.(c) Experimental setup including key hyperparameters and dataset splits.

(a) Main experimental results on classification tasks across different sub-tasks for in-domain (seen during training) and out-of-domain (unseen during training) categories, without out-of-domain severity. Numbers marked with ∗ correspond to the True Positive Rate (TPR) rather than accuracy (ACC).								
Method Type	Model	In-domain(ACC)				Out-of-domain(ACC)		
		Plant	Disease	Severity	All	Plant	Disease	All
Supervised	ResNet18 [36]	95.8	90.6	86.1	77.9	71.0	56.6	43.3
	ResNet34 [36]	95.6	90.4	85.1	78.3	71.5	56.6	43.5
	ResNet50 [36]	95.7	90.1	85.8	77.6	71.1	57.6	43.9
	ShuffleNet [37]	97.3	92.4	84.7	79.9	79.8	64.8	50.0
	ViT [38]	20.9	8.3	32.8	7.5	0.0	0.0	0.0
	DenseNet169 [39]	96.8	91.4	85.1	79.6	76.8	61.4	47.1
	DenseNet264 [39]	96.5	91.3	85.5	79.3	76.8	61.8	47.9
	RegNet [40]	94.5	90.3	84.2	79.2	69.3	56.6	44.2
	ConvNeXt [41]	95.5	89.8	84.4	77.8	70.9	57.6	45.5
	EfficientNet [42]	97.0	92.9	87.4	81.0	78.9	63.7	49.8
	EfficientNetV2 [42]	97.3	93.2	87.8	81.3	79.4	64.0	50.0
	MobileNetV2 [43]	97.2	92.3	85.5	79.2	81.5	67.0	49.3
	MobileNetV3 [44]	97.2	92.4	85.5	79.0	79.5	64.5	47.2
	TransNeXt [45]	96.0	87.7	83.7	75.4	77.2	59.1	45.3
	OverLoCK [46]	92.4	82.8	81.4	72.8	73.1	53.6	43.2
	Ours	**98.6**	**94.9**	**90.6**	**82.8**	**84.7**	**70.1**	**54.7**
Open-Set	PALM [47]	61.0	57.0	44.1	49.0	59.8∗		
	DHE [48]	51.2	47.0	46.2	42.1	50.5∗		
	DOS [49]	93.8	88.9	73.5	74.9	54.5∗		
	NegCosSch [50]	73.1	54.1	64.9	43.9	49.5∗		
	Ours	**98.6**	**94.9**	**90.6**	**82.8**	**82.1∗**		

(b) Statistical validation of the strongest baselines.			
Model	In-domain (All ACC)	Out-of-domain (All ACC)	Open-set (TPR)
EfficientNetV2	81.3 ± 0.4	50.0 ± 0.5	–
*p*-value (vs Ours)	0.0059	0.0006	–

DOS	74.9 ± 0.6	–	54.5 ± 0.7
*p*-value (vs Ours)	–	–	0.0001

Ours	**82.8** ± **0.2**	**54.7** ± **0.3**	**82.1** ± **0.4**

(c) Experimental setup.			
Param	Val	Param	Val
Input	256× 256	Opt	SGD
Patch	16× 16	Init LR	0.0004
Stride	11	*τ*_*s*_	0.1
Act	GELU	*τ*_*t*_	0.04

Hyperparameters			

Subset	Train	Val	Test
CKL	89031	11003	9956
CDA	17177	–	–
Unseen	–	–	5042

Dataset splits			

To validate the effectiveness of our proposed method, we conduct a series of comparative experiments including conventional supervised learning approaches such as ResNet [36], ShuffleNet [37], ViT [38], DenseNet [39], RegNet [40], ConvNeXt [41], EfficientNet [42], and MobileNet [43], as well as state-of-the-art open-set methods including PALM [47], DHE [48], DOS [49] and NegCosSch [50].

### 3.2 Dataset composition and splits

To support the two-stage training strategy and final evaluation protocol, the dataset is divided into three subsets corresponding to the Common Knowledge Learner (CKL) stage, the Cross-Domain Adapter (CDA) stage, and unseen test evaluation. These subsets simulate scenarios where models are trained on well-annotated common categories, adapted using unlabeled rare categories, and evaluated on unseen plant–disease combinations. Image statistics for each subset are summarized in Table 1c.

**CKL Subset (In-Domain Training).** This subset contains 19 plant species, 63 disease categories, and four severity levels. All images are annotated and shared across three task branches: plant classification, disease recognition, and severity grading. The CKL subset serves as the primary supervised training set for learning representations of plant species, disease categories, and severity levels.

**CDA Subset (Out-of-Domain Adaptation).** This subset is drawn from the Leaf-CG dataset and contains images from rare plant disease categories, defined as plant–disease classes with fewer than 100 samples. It includes 32 plant species and 153 disease categories. Although annotations exist, these labels are discarded during training and the subset is used in the CDA stage to simulate an unlabeled adaptation setting.

**Unseen Test Set.** The evaluation set includes 21 novel plant species and 34 novel disease categories not present in the CKL subset. This setting enables evaluation of the model's generalization ability on unseen plant–disease combinations.

### 3.3 Quantitative comparisons with the baselines

To validate the effectiveness of our method, we compare it against a series of baseline methods, including supervised learning–based methods and open-set recognition methods, as shown in Table 1a. For supervised learning–based methods, we compare our model to EfficientNetV2, ConvNeXt, and MobileNetV3, trained with both in-domain and out-of-domain labels. Our model achieves the highest in-domain ACC of 82.8% and out-of-domain ACC of 54.7%, surpassing the next best results of 81.3% and 50.0%. This improvement stems from the two-stage framework combining CKL and MLC, enabling transferable feature learning across plant species, disease types, and severity grading. Compared with EfficientNetV2, we also achieve higher ACC in plant classification (98.6%), disease classification (94.9%), and severity grading (90.6%). While MobileNetV2 suffers a notable drop in out-of-domain ACC (from 79.2% to 49.3%), our model maintains robust performance through multi-level contrastive learning and library-based retrieval. Overall, it outperforms baselines in both in-domain and out-of-domain tasks, demonstrating strong generalization and fine-grained feature representation.

Regarding in-domain categories, our model achieves strong performance, reaching 82.8% ACC and surpassing PALM (49.0%), DHE (42.1%) and DOS (74.9%). This performance benefits from multi-level contrastive learning and library-based retrieval. For out-of-domain categories, our model attains the highest TPR of 82.1%, outperforming PALM (59.8%), DHE (50.5%) and DOS (54.5%). Effective cross-domain adaptation ensures this improvement, enhancing the model's ability to distinguish between in-domain and out-of-domain categories. Overall, our method demonstrates strong in-domain accuracy while substantially improving TPR on out-of-domain categories.

We repeat all primary experiments three times with different random seeds and report the mean and standard deviation in Table 1b. We also perform paired t-tests against the strongest baseline to verify that improvements are statistically significant (p < 0.05).

### 3.4 Qualitative comparisons with the baselines

We present a qualitative comparison of our method with baseline models using heatmap visualizations (Fig. 5a). For plant classification, our model focuses on the entire leaf, whereas baselines often exhibit incomplete attention. In disease recognition, it accurately highlights lesion locations and severity, while baselines tend to diffuse attention across the leaf or irrelevant regions. For severity grading, our approach captures the full leaf structure, essential for assessing lesion proportion, in contrast to baselines that focus on local lesions or background. These results indicate that our method produces attention maps better aligned with the diagnostic requirements across tasks.

### 3.5 Impact of backbone architectures

To assess the impact of backbone architecture on CKL performance, we conduct an ablation study comparing seven representative feature extractors. We evaluate these architectures across three tasks, plant classification, disease recognition, and severity grading, on both in-domain and out-of-domain test sets. In this section, ViT refers to the backbone architecture integrated within the CKL framework unless otherwise stated.

**Fig. 6:**
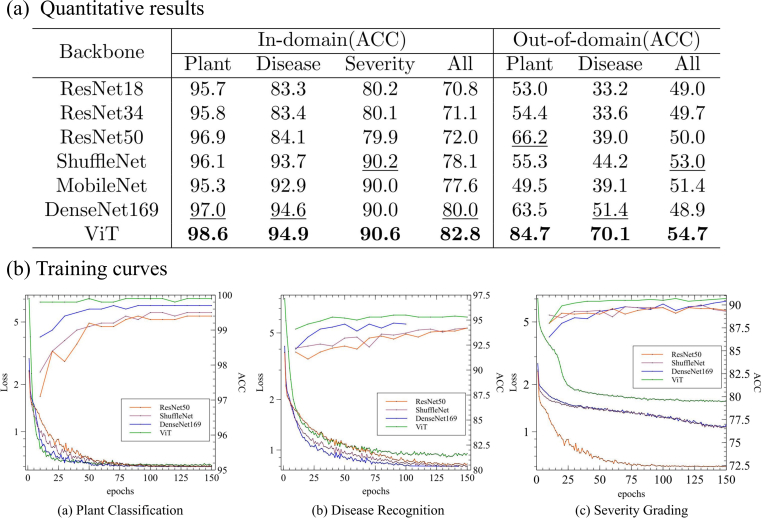
Comparison of different backbone architectures in terms of quantitative performance and training dynamics. (a) Quantitative evaluation results of different backbone networks on in-domain and out-of-domain tasks, including plant classification, disease recognition, and severity grading. (b) Training curves of representative backbone networks (ResNet50, ShuffleNet, DenseNet169, and ViT) on three subtasks: plant classification, disease recognition, and severity grading, showing the evolution of training loss (left axis) and accuracy (right axis) over epochs.

First, all models perform well on in-domain data, with accuracy increasing from ResNet18 to DenseNet169, and ViT outperforms all others across tasks, achieving the highest overall accuracy, as shown in Fig. 6a. Second, when evaluated on out-of-domain data, ViT demonstrates a substantial advantage. This consistent performance highlights the transformer-based architecture's strong ability to generalize across domains, confirming the effectiveness of using ViT as the backbone in our CKL module.

Fig. 6b shows the training curves of representative backbones across three tasks: plant classification, disease recognition, and severity grading. For plant classification, ViT consistently achieves higher accuracy and lower training losses compared to other models, which indicates faster convergence. In disease recognition, ViT again outperforms all models, which shows a steeper loss reduction and higher ACC. For severity grading, ViT maintains the best performance, with the highest ACC and lowest loss, while other models, especially ResNet50, show slower convergence and higher losses. These results highlight the effectiveness of ViT, indicating that transformer-based architectures converge efficiently and offer strong feature representations suitable for our CKL module.

### 3.6 Impact of cross-domain fine-tuning

**Fig. 7:**
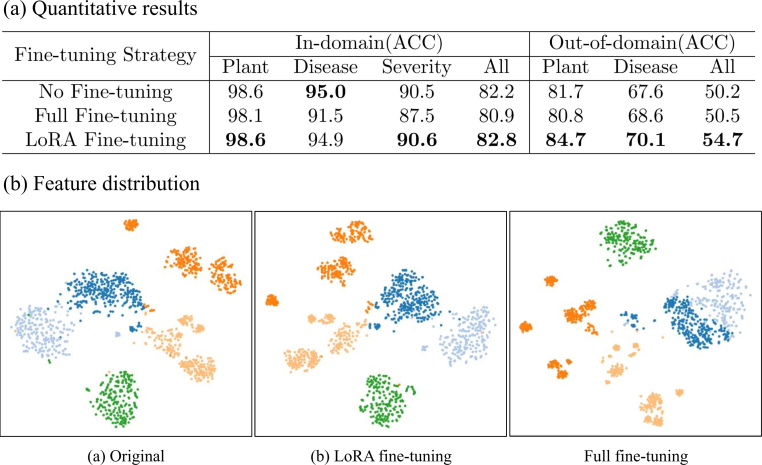
Comparison of different fine-tuning strategies in terms of quantitative performance and feature representation quality. (a) *Quantitative results* of different fine-tuning strategies under in-domain and out-of-domain settings, covering plant classification, disease recognition, and severity grading. (b)*Feature distribution* under different fine-tuning strategies in the Cross-domain Adapter stage: (a) original features without fine-tuning, (b) features after LoRA fine-tuning, and (c) features after full fine-tuning. The visualizations are based on randomly selected samples from five out-of-domain categories. Compared to the original and fully fine-tuned settings, LoRA fine-tuning produces more compact and discriminative clusters, indicating improved cross-domain representation adaptation.

To assess the effectiveness of the CDA, we conduct an ablation study on different fine-tuning strategies during out-of-domain adaptation. We compare no fine-tuning, full fine-tuning, and LoRA fine-tuning, which updates only a subset of parameters. As shown in Fig. 7a, LoRA fine-tuning achieves the best overall performance, with in-domain ACC reaching 82.8% and out-of-domain ACC 54.7%, demonstrating an effective balance between retaining prior knowledge and adapting to new data.

Furthermore, to explore how different fine-tuning strategies influence the feature space distribution, we use t-SNE to visualize the learned representations, as shown in Fig. 7b. LoRA fine-tuning results in more compact and discriminative clusters, indicating better cross-domain representation adaptation. In contrast, full fine-tuning compresses the clusters further but introduces issues: clusters of the same class split into multiple sub-clusters, and the boundaries between different classes become less distinct. In summary, LoRA fine-tuning preserves class separation and is computationally more efficient, making it the optimal choice for cross-domain adaptation without sacrificing performance.

### 3.7 Impact of contrastive library size

To evaluate the effect of contrastive library size on model performance, we conduct an ablation study by reducing the number of samples per category at ratios of 30%, 60%, and 90%. As shown in Table 2a, decreasing the library size generally leads to lower performance, with out-of-domain tasks particularly affected. The most pronounced declines occur in disease recognition and full-category evaluation, reflecting their sensitivity to fewer comparison samples. These results highlight the importance of maintaining a diverse and sufficiently large contrastive library to provide robust reference features and enhance generalization to unseen domains.

**Table 2:** Comprehensive evaluation of the proposed contrastive-library-based framework under different experimental settings. (a) Ablation study on the size of the contrastive library, analyzing its impact on both in-domain and out-of-domain performance. (b) Generalization results on entirely unseen plant and disease categories using newly constructed contrastive libraries. (c) Performance comparison under different label supervision strategies, evaluating the effectiveness of the proposed two-stage method in scenarios with limited labeled out-of-domain data.

(a) Effect of contrastive library size reduction on in-domain and out-of-domain performance.							
Reduction	In-domain(ACC)				Out-of-domain(ACC)		
	Plant	Disease	Severity	All	Plant	Disease	All
−30%	**99.7**	**96.8**	**88.3**	**84.8**	**82.5**	**68.4**	**52.4**
−60%	99.5	95.1	84.9	81.7	81.7	66.7	50.7
−90%	99.4	94.4	80.8	77.1	75.3	58.9	44.0

(b) Generalization performance(ACC) on entirely novel plant and disease categories.				
Method	Plant	Disease	Severity	All
Ours (Stage I Model)	99.5	100.0	70.0	62.7
Ours (Fully Fine-Tuned Model)	93.1	96.7	**76.7**	59.9
Ours (LoRA Fine-Tuning Model)	**99.6**	**100.0**	70.0	**64.5**

(c) Comparison of different label supervision strategies under in-domain and out-of-domain settings.							
Setting	In-domain(ACC)				Out-of-domain(ACC)		
	Plant	Disease	Severity	All	Plant	Disease	All
Ours (All Labels)	**98.9**	**95.6**	90.4	**84.7**	**90.5**	**74.3**	**59.9**
Ours (In-domain Labels)	98.6	94.9	**90.6**	82.8	84.7	70.1	54.7

### 3.8 Effect of stage-wise fine-tuning on generalization to unseen categories

To evaluate generalization to entirely novel categories, we test the model on plant and disease types unseen during both Stage I and Stage II. As shown in Table 2b, the Stage I model already achieves high Plant (99.5%) and Disease (100.0%) accuracies, with reasonable Severity (70.0%) and overall score (62.7%). Full fine-tuning improves Severity (76.7%) but reduces Plant (93.1%) and Disease (96.7%) accuracies, leading to a lower overall score (59.9%). In contrast, LoRA fine-tuning maintains near-perfect Plant (99.6%) and Disease (100.0%) accuracies while preserving Severity (70.0%), achieving the highest overall score (64.5%). These results demonstrate that our stage-wise training combined with LoRA adaptation effectively balances performance across tasks, preserving foundational knowledge while generalizing to unseen plant and disease categories.

### 3.9 Effect of label supervision on out-of-domain data

To evaluate the model's performance, we compare a fully supervised setup, where both in-domain and out-of-domain data are annotated and used during training (ours (all labels)), with our two-stage approach, where only in-domain annotations are available and out-of-domain data are adapted through unlabeled distribution alignment (ours (in-domain labels)). The results are shown in Table 2c. As expected, the fully supervised setup achieves the highest accuracy on most tasks, serving as a performance upper bound. Our two-stage method, without any out-of-domain labels, delivers comparable results: overall performance on out-of-domain data drops only from 59.9% to 54.7%. This small gap demonstrates the effectiveness of our domain-aware adaptation strategy and confirms that the framework generalizes well under limited label availability.

### 3.10 Computational efficiency and inference

To evaluate the computational efficiency of our model, we measure the inference time and memory footprint on a test set consisting of 15,007 test images against a contrastive library containing 93,482 images. Average inference time per image*:* 0.00977 s. GPU peak memory usage*:* 1067.38 MB. CPU RAM usage*:* 8959.76 MB. These results demonstrate that the proposed method maintains efficient inference performance under a large-scale setting. The GPU memory consumption remains moderate for modern hardware platforms, while the CPU memory usage mainly arises from feature storage and similarity matrix computation. Overall, these metrics indicate the practical feasibility and scalability of our approach for real-world deployment.

## 4 Discussion

### 4.1 Capability of the proposed multi-task framework

The framework employs a unified network architecture with consistent data input, while training each task independently using task-specific labels for plant classification, disease classification, and severity grading. This approach generates separate weights for each task, ensuring task-specific reasoning without cross-task interference. By maintaining architectural consistency and leveraging contrastive learning, the framework can be easily extended to new plant species and disease categories by updating the label space, supporting robust and scalable deployment in diverse smart agriculture scenarios.

### 4.2 Extended use cases and model capabilities

The framework demonstrates strong universality and scalability across diverse applications. By leveraging instance-level comparison rather than class probabilities alone, it effectively handles long-tail data distributions and improves recognition of rare categories. It also supports open-set identification, allowing the system to operate beyond closed-set assumptions. The use of contrastive prediction enhances interpretability, as each decision can be traced to the most similar labeled examples, providing transparency and credibility. Furthermore, the framework enables controllable task expansion: updating the comparison feature library allows adaptation to new categories without modifying the network architecture.

**Fig. 8:**
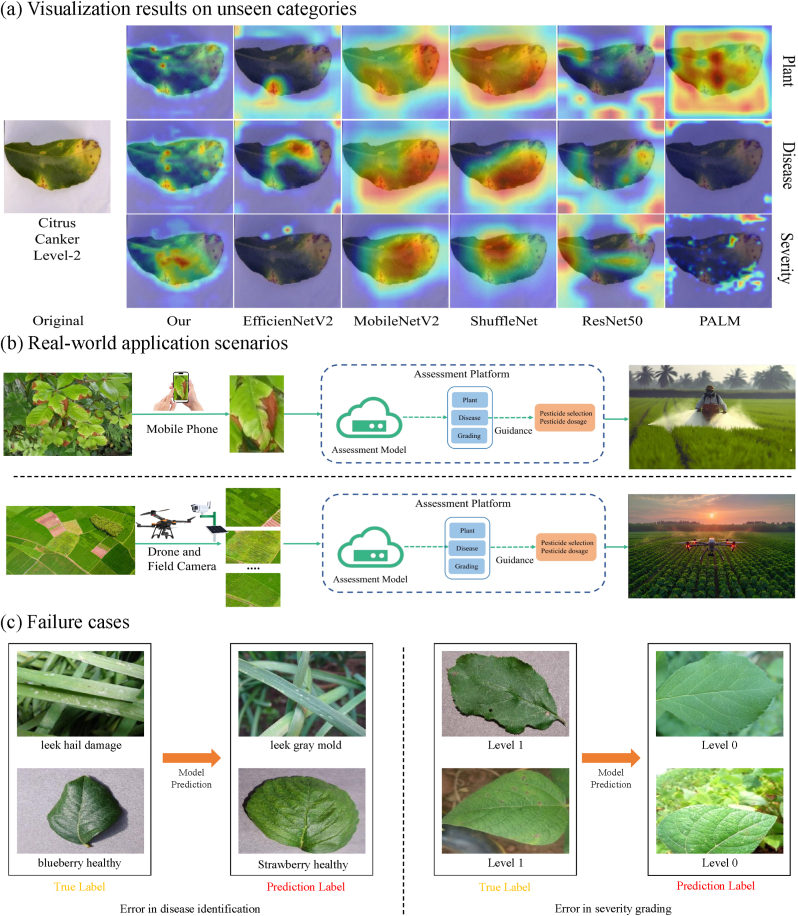
Qualitative results and real-world applications of the proposed plant disease assessment framework. (a) Visualization results on previously unseen categories, comparing our method with representative baseline models (EfficientNetV2, MobileNetV2, ShuffleNet, ResNet50 and PALM) across three tasks: plant species classification, disease identification, and severity grading. (b) Real-world application scenarios of the proposed framework. The upper branch illustrates an automated guidance pipeline, where leaf images captured by mobile devices are analyzed to provide plant, disease, and severity assessments for precise pesticide recommendations. The lower branch presents a smart pesticide management system, in which drone- and camera-acquired field images are processed to enable large-scale, real-time disease diagnosis and intelligent pesticide application.Representative failure cases illustrating typical errors in disease identification and severity grading.

Consistent with qualitative observations on in-training categories (Fig. 5), visualizations on unseen categories (Fig. 8a) show similar task-aligned attention patterns. The framework preserves its ability to focus on global leaf structures for plant classification, localized lesions for disease recognition, and both leaf context and lesion extent for severity grading. This consistency across in-distribution and out-of-distribution settings indicates that the learned representations generalize beyond the training distribution.

### 4.3 Limitations of the proposed framework

Although our method proves effective, several minor limitations remain. First, the reliance on high-quality contrast libraries means that instability in sample labeling or image quality may slightly affect performance, although such influence is often mitigated by the robustness of contrastive learning. Second, the need to train multiple task-specific weights separately may introduce some complexity in model management, though this does not substantially affect inference efficiency. Finally, as with most data-driven classification systems, extremely rare or unseen disease categories can still pose challenges. Overall, however, these limitations do not diminish the practical value or scalability of the proposed framework.

### 4.4 Overcoming limitations of existing methods

Recent deep learning studies have significantly improved the accuracy of plant disease recognition; however, several limitations remain when deploying these models in real-world agricultural scenarios. First, many existing approaches rely on fixed classification heads and predefined label spaces, which restrict their ability to handle previously unseen plant–disease combinations. When novel categories appear, these models often misclassify them or assign them to a generic unknown class. In contrast, the proposed framework replaces the conventional classification head with a contrastive feature library and similarity-based inference mechanism. This design enables the label space to be expanded simply by adding representative samples to the library, allowing the model to recognize new categories without retraining.

Second, previous studies typically focus on disease identification alone, while severity grading is less frequently addressed despite its importance for practical disease management. Our framework jointly models plant species recognition, disease classification, and severity grading within a unified architecture, enabling comprehensive plant health assessment.

Third, model performance in existing studies often degrades under cross-domain conditions due to variations in imaging devices, illumination, and background complexity. To address this issue, the proposed Cross-Domain Adapter leverages unlabeled out-of-domain data through a teacher–student learning strategy with LoRA-based adaptation, enabling effective feature alignment across domains. Together, these designs improve the robustness and scalability of plant disease recognition systems in realistic agricultural environments.

### 4.5 Applications

The proposed multi-task learning framework based on contrastive feature libraries shows great potential for intelligent management and control of plant diseases. As illustrated in Fig. 8b upper branch, farmers can capture images of diseased leaves using mobile devices and upload them to the diagnostic platform. The model then identifies the crop variety, disease type, and severity level, and provides precise recommendations on pesticide selection and dosage based on the diagnostic results, thereby enabling effective and environmentally friendly disease control.

In large-scale farmland settings, as shown in Fig. 8b lower branch, our framework can be seamlessly integrated with unmanned aerial vehicles (UAVs) and fixed monitoring cameras to automatically capture and transmit field images to the diagnostic platform. The system conducts real-time disease analysis and generates crop- and region-specific pesticide application strategies. This not only reduces the reliance on labor-intensive field inspections but also supports the automation of intelligent agricultural machinery, improving efficiency, lowering operational costs, and enabling precision and environmentally sustainable crop protection at scale.

### 4.6 Failure case analysis

To further analyze the robustness and limitations of our model, we present representative failure cases for both disease identification and severity grading in Fig. 8c. These examples provide insight into scenarios where the model may produce incorrect predictions and help reveal the underlying challenges of plant disease recognition under complex visual conditions.

For disease identification, misclassifications mainly occur between visually similar disease categories that share highly overlapping texture patterns, lesion distributions, and color characteristics. When symptom appearance differences are subtle, the model may produce incorrect predictions despite strong overall performance. This observation suggests that fine-grained inter-class discrimination remains a challenging problem in plant disease recognition.

For severity grading, most errors occur between adjacent severity levels (e.g., Level-0 and Level-1). The visual differences between neighboring severity stages are often subtle and may be further influenced by illumination variations, background clutter, or partial occlusion. These factors increase intra-class variability and reduce the separability between nearby severity categories. Overall, these failure cases indicate that while the proposed method achieves strong overall performance, further improvements could be obtained by enhancing fine-grained feature representation and improving robustness to environmental variations.

### 4.7 Comparison with knowledge-driven domain adaptation methods

Knowledge-driven domain adaptation has been explored in different forms to mitigate domain shift by introducing prior or external knowledge into the adaptation process. For example, prior-knowledge-guided UDA methods [51] use target class-distribution priors, such as unary bounds and binary relationships, to rectify pseudo labels during self-training. KDDA-Balance [52] incorporates gait-related prior indicators into adversarial alignment and dual-classifier discrepancy learning for cross-age balance assessment. These studies demonstrate that knowledge can improve domain adaptation, but the role of knowledge is usually formulated as a label-space constraint, a hand-crafted domain-specific feature guide, or an auxiliary signal for distribution alignment. In contrast, our framework does not assume target class-distribution priors or manually designed disease phenotype indicators. Instead, the Cross-Domain Adapter uses unlabeled out-of-domain plant disease images to refine the learned visual representation through teacher–student learning and LoRA-based parameter-efficient tuning.

The EasyDAM series is particularly relevant because it also addresses agricultural domain adaptation and automatic labeling. EasyDAM [53] transfers labels across fruit species through CycleGAN-based image translation and pseudo-label self-learning, while EasyDAM_V2 [54] improves cross-species translation for fruits with partial shape differences using Across-CycleGAN and adaptive pseudo-label thresholding. EasyDAM_V3 [19] further introduces a multidimensional phenotypic feature space based on shape, color, and texture to select the optimal source domain and constructs synthetic target-domain data with knowledge-graph-guided synthesis rules. EasyDAM_V4 [55] extends this line by using Guided-GAN and multidimensional phenotypic constraints to handle fruits with significant shape differences. Overall, the EasyDAM series mainly performs data-side adaptation, where knowledge supports source-domain selection, source-to-target image generation, and synthetic target-domain data construction for detector training or automatic labeling. By contrast, our method performs model-side adaptation: Stage II Cross-Domain Adapter(CDA) updates model representations with unlabeled out-of-domain plant disease images, while the contrastive library supports inference-time similarity matching and label-space expansion. Therefore, although our work shares the broad motivation of knowledge-driven domain adaptation, it differs from EasyDAM and other representative methods in the form of knowledge, the stage at which knowledge intervenes, and the final recognition objective.

## 5 Conclusion

In this study, we present a knowledge-driven, unified multi-task framework for plant disease classification and severity grading, designed to address challenges such as domain shift and the identification of unseen disease categories. The increasing complexity of plant disease monitoring in real-world settings makes this approach highly relevant, offering a solution that adapts seamlessly to new unseen disease types.

Our framework employs a dual-branch contrastive design, which captures both global semantic context and fine-grained lesion cues, improving the model's generalization capabilities across various species and diseases. The modular architecture allows for easy adaptation to species classification, disease identification, and severity grading, requiring only updates to the label schema and task-specific weights, without the need to alter the network structure. This ensures scalability and practical deployment in dynamic agricultural environments.

Through rigorous evaluation on the curated Leaf-CG dataset, consisting of approximately 441,488 annotated images across diverse species and diseases, our framework demonstrates state-of-the-art accuracy and strong cross-domain generalization. The results, including improvements in True Positive Rate (TPR) and overall accuracy, show its ability to handle out-of-domain conditions effectively, making it a scalable solution for intelligent plant health monitoring. This study paves the way for future advancements in open-set recognition, long-tail category adaptation, and interpretable AI-based plant disease diagnosis.

## Authors’ contributions

Zhihao Yuan: be responsible for method design, writing, revising papers, drawing charts, and revision of articles. Xingcai Wu: put forward the main research problems of this paper, control the writing progress of the paper, and participate in the revision of the paper. Yang Liu: participated in the construction of data sets. Xiaomin Shuai: participated in the construction of data sets. Xulong Huang: participated in the construction of data sets. R.D.S.M Gunarathna: reviewed the manuscript. Peijia Yu: reviewed the manuscript. Yuanyuan Xiao: reviewed the manuscript. Qi Wang: reviewed the manuscript.

## Declaration of competing interest

The authors declare that they have no known competing financial interests or personal relationships that could have appeared to influence the work reported in this paper.

## Data Availability

The data, code, and model weights used in this paper can be downloaded from: https://www.uniplantcg.samlab.cn. This paper uses the Leaf-CG dataset, which is constructed based on an ongoing research project conducted by our team. The test subset of the Leaf-CG dataset is publicly available on the website. In addition, three types of model weights are involved in this paper: plant.pth contains the trained parameters of the plant classification network, disease.pth corresponds to the trained parameters of the plant disease recognition network, and severity.pth provides the trained parameters of the disease severity assessment network. The inference process of the models has been explained on the website.
