# Supplementary material for: A knowledge-driven unified framework for plant disease classification and severity grading via domain adaptation
Source: Plant Phenomics. 2026 Jun 6;8(3):100233. doi: 10.1016/j.plaphe.2026.100233 (PMC13276328; doi:10.1016/j.plaphe.2026.100233)
Supplement: Multimedia component 1 [file mmc1.pdf]

Supplemental Material

1 Mathematical formulation of the proposed framework.

To further clarify the mechanism of the proposed framework, we provide a formal formulation of its three main components: supervised representation learning, unlabeled model-side cross-domain adaptation, and contrastive-library-based inference.

Step 1: Supervised representation learning on labeled common categories. Let

$$\mathcal{D}_L = \{(x_i, y_i)\}_{i=1}^{N} \tag{1}$$

denote the labeled training set from common plant and disease categories, where x_i is an input image and y_i is the corresponding task label. For different tasks, y_i can represent plant species, disease category, or severity level. Let $f_\theta(\cdot)$ denote the feature encoder parameterized by θ. The feature representation of an input image is

$$z_i = f_\theta(x_i). \tag{2}$$

In Stage I, the model is trained using a supervised classification loss and a metric-learning loss:

$$\mathcal{L}_{\text{StageI}} = \mathcal{L}_{\text{cls}} + \lambda_{\text{tri}}\mathcal{L}_{\text{tri}}, \tag{3}$$

where $\mathcal{L}_{\text{cls}}$ is the supervised classification loss, $\mathcal{L}_{\text{tri}}$ is the triplet loss, and λ_{tri} is a balancing coefficient. The triplet loss is written as

$$\mathcal{L}_{\text{tri}} = \sum_i \left[m + d(z_i, z_i^+) - d(z_i, z_i^-)\right]_+, \tag{4}$$

where z_i^+ and z_i^- denote positive and negative samples relative to z_i, $d(\cdot,\cdot)$ is a distance metric, m is the margin, and $[\cdot]_+ = \max(\cdot, 0)$. The initial parameters learned in Stage I are therefore

$$\theta^{(0)} = \arg\min_\theta \mathcal{L}_{\text{StageI}}(\mathcal{D}_L; \theta). \tag{5}$$

Step 2: Model-side cross-domain adaptation with unlabeled data. Let

$$\mathcal{U} = \{u_j\}_{j=1}^{M} \tag{6}$$

denote the unlabeled out-of-domain plant disease images. Starting from $\theta^{(0)}$, the Cross-Domain Adapter adapts the representation to out-of-domain data without requiring predefined target-domain categories or manual labels.

For a learnable weight matrix W in the model, the LoRA-based parameter-efficient update is formulated as

$$W^* = W^{(0)} + \Delta W, \quad \Delta W = BA, \tag{7}$$

where A and B are low-rank trainable matrices. Let ϕ_s denote the trainable LoRA parameters of the student model. The adapted student parameters can be represented as

$$\theta_s = \theta^{(0)} \oplus \Delta\phi_s, \tag{8}$$

where $\oplus$ denotes inserting LoRA updates into the original model parameters.

A teacher model is maintained by exponential moving average (EMA). Let $\Delta\phi_t$ and $\Delta\phi_s$ denote the LoRA parameters of the teacher and student models, respectively. The teacher update is

$$\Delta\phi_t \leftarrow \mu\Delta\phi_t + (1-\mu)\Delta\phi_s, \tag{9}$$

where μ is the EMA momentum.

For an unlabeled image u_j, the teacher and student output distributions are denoted as

$$P_t(u_j) = \mathrm{softmax}\left(q_{\theta_t}(a_t(u_j))/\tau\right), \tag{10}$$

$$P_s(u_j) = \mathrm{softmax}\left(q_{\theta_s}(a_s(u_j))/\tau\right), \tag{11}$$

where $q_\theta(\cdot)$ denotes the prediction function, $a_t(\cdot)$ and $a_s(\cdot)$ denote teacher and student augmentations, and τ is the temperature parameter. The cross-domain adaptation objective is formulated as a teacher–student consistency loss:

$$\mathcal{L}_{\mathrm{CDA}} = \frac{1}{M}\sum_{j=1}^{M} \mathrm{KL}\left(\mathrm{sg}[P_t(u_j)] \| P_s(u_j)\right), \tag{12}$$

where $\mathrm{sg}[\cdot]$ denotes the stop-gradient operation. The adapted LoRA parameters are obtained by

$$\phi_s^* = \arg\min_{\phi_s} \mathcal{L}_{\mathrm{CDA}}(\mathcal{U}; \theta^{(0)}, \phi_s), \tag{13}$$

and the final adapted encoder is

$$\theta^* = \theta^{(0)} \oplus \Delta\phi_s^*. \tag{14}$$

Thus, the cross-domain adaptation component of the proposed framework is an unlabeled representation-alignment process in parameter space.

Step 3: Contrastive-library-based instance-level inference. After obtaining the adapted encoder $f_{\theta^*}(\cdot)$, prediction is performed through a contrastive feature library rather than a fixed classification head. Let the contrastive library be

$$\mathcal{C} = \{(r_k, y_k)\}_{k=1}^{K}, \tag{15}$$

where r_k is a labeled reference image and y_k is its corresponding label. The feature of a query image x and the feature of a reference image r_k are

$$z_x = f_{\theta^*}(x), \quad z_k = f_{\theta^*}(r_k). \tag{16}$$

The similarity between x and r_k is computed by combining global and local similarity:

$$s(x, r_k) = \alpha s_{\mathrm{global}}(x, r_k) + (1-\alpha)s_{\mathrm{local}}(x, r_k), \tag{17}$$

where α controls the contribution of the global and local terms. The global similarity can be defined as cosine similarity:

$$s_{\mathrm{global}}(x, r_k) = \frac{z_x^\top z_k}{\|z_x\|_2 \|z_k\|_2}. \tag{18}$$

The local similarity term measures fine-grained correspondence between local feature regions:

$$s_{\text{local}}(x, r_k) = \frac{1}{P} \sum_{p=1}^{P} \max_q \frac{h_x^{p\top} h_k^q}{\|h_x^p\|_2 \|h_k^q\|_2}, \tag{19}$$

where h_x^p and h_k^q denote local patch features of the query and reference images, respectively.

For a label y, the class-level score is obtained by aggregating the similarities of reference samples belonging to that label:

$$S_y(x) = \max_{k: y_k = y} s(x, r_k). \tag{20}$$

The final prediction is

$$\hat{y} = \arg\max_{y \in \mathcal{Y}_\mathcal{C}} S_y(x), \tag{21}$$

where $\mathcal{Y}_\mathcal{C}$ denotes the label set contained in the contrastive library.

Step 4: Label-space expansion without backbone retraining. The above formulation shows that model-side adaptation and inference-side label-space expansion are separated. The adapted encoder is obtained by

$$\theta^* = F_{\text{adapt}}(\theta^{(0)}, \mathcal{U}), \tag{22}$$

whereas inference is performed by

$$\hat{y} = F_{\text{inf}}(x, \mathcal{C}, \theta^*). \tag{23}$$

The contrastive library $\mathcal{C}$ does not participate in the Stage II adaptation objective:

$$\mathcal{L}_{\text{CDA}} = \mathcal{L}_{\text{CDA}}(\mathcal{U}; \theta^{(0)}, \phi_s), \quad \mathcal{C} \notin \mathcal{L}_{\text{CDA}}. \tag{24}$$

Therefore, adding a new category only requires extending the reference library:

$$\mathcal{C}' = \mathcal{C} \cup \{(r_{\text{new}}, y_{\text{new}})\}. \tag{25}$$

The adapted encoder remains unchanged:

$$\theta^*_{\mathcal{C}'} = \theta^*_{\mathcal{C}}. \tag{26}$$

The new prediction rule becomes

$$\hat{y}' = F_{\text{inf}}(x, \mathcal{C}', \theta^*). \tag{27}$$

This demonstrates that new plant disease categories or severity labels can be incorporated by updating the reference library rather than retraining the backbone. Therefore, the proposed framework separates model-side cross-domain adaptation from inference-side label-space expansion.
